# Aztreonam/avibactam activity against Enterobacterales from European medical centres: summary of 5 years of surveillance prior to approval for clinical use (2019–2023)

**DOI:** 10.1093/jac/dkaf161

**Published:** 2025-06-16

**Authors:** Helio S Sader, John H Kimbrough, Marisa L Winkler, Mariana Castanheira, Rodrigo E Mendes

**Affiliations:** Element Iowa City (JMI Laboratories), 345 Beaver Kreek Centre, Suite A, North Liberty, IA 52317, USA; Element Iowa City (JMI Laboratories), 345 Beaver Kreek Centre, Suite A, North Liberty, IA 52317, USA; Element Iowa City (JMI Laboratories), 345 Beaver Kreek Centre, Suite A, North Liberty, IA 52317, USA; Element Iowa City (JMI Laboratories), 345 Beaver Kreek Centre, Suite A, North Liberty, IA 52317, USA; Element Iowa City (JMI Laboratories), 345 Beaver Kreek Centre, Suite A, North Liberty, IA 52317, USA

## Abstract

**Objectives:**

To evaluate the *in vitro* activity of aztreonam/avibactam against Enterobacterales from European medical centres during the 5-year period prior to its approval for clinical use in Europe.

**Methods:**

Thirty thousand seventy-four Enterobacterales isolates were consecutively collected in 2019–2023 from 19 medical centres in Eastern Europe and Mediterranean region (E-EU; *n* = 8074) and 27 medical centres in Western Europe (W-EU; *n* = 22 000) and susceptibility tested by broth microdilution. Carbapenem-resistant Enterobacterales (CRE) and isolates with elevated MICs (>4 mg/L) for aztreonam/avibactam were molecularly characterized.

**Results:**

Aztreonam/avibactam was active against 99.8% and >99.9% of Enterobacterales from E-EU and W-EU, respectively and exhibited potent activity against CRE isolates (MIC_50/90_, 0.25/0.5 mg/L; 99.6%/99.7% susceptible in E-EU/W-EU). Cefiderocol was active against 74.8%/87.6% of CREs from E-EU/W-EU. Ceftazidime/avibactam, meropenem/vaborbactam, and imipenem/relebactam retained moderate activity against CRE isolates from W-EU (68.3–80.3% susceptibility) but showed limited activity against CRE isolates from E-EU (45.1–63.0% susceptible). The occurrence of carbapenemases varied markedly among the countries evaluated. In general, the MBLs predominated in E-EU and the KPCs prevailed in W-EU. Decreased susceptibility to aztreonam/avibactam was predominantly due to PBP3 alterations and production of CMY and/or CTX-M β-lactamases among *Escherichia coli*, and hyperexpression of *ampC* plus porin alterations in *Enterobacter cloacae* species complex and *Klebsiella aerogenes*.

**Conclusions:**

The results of this investigation provide a valuable benchmark for monitoring the *in vitro* activity of aztreonam/avibactam after its clinical approval in Europe and emphasizes the importance of comprehensive surveillance programmes to monitor the emergence of high-risk clones and resistance mechanisms to newly approved antimicrobial agents.

## Introduction

Carbapenem-resistant Enterobacterales (CRE) are a major global health problem that seriously compromises the treatment of healthcare-associated infections.^1^ Infections caused by CRE are associated with elevated mortality rates, mainly due to the delay prior to administering effective antimicrobial therapy.^2^

The most clinically important mechanism of carbapenem resistance in Enterobacterales is hydrolysis by carbapenemase enzymes. Although *Klebsiella pneumoniae* carbapenemase (KPC) is the most common carbapenemase worldwide, other carbapenemases such as oxacillinase (OXA)-48-like and the metallo-β-lactamases (MBLs) NDM, VIM, and IMP play an important role in some geographic areas.^3,4^ Moreover, non–carbapenemase-producing CRE may also account for an important proportion of CRE isolates, and the common mechanism of carbapenem resistance in these isolates is usually a combination of a β-lactamase [extended-spectrum β-lactamases (ESBLs) or AmpC] and mutations in the outer membrane porins.^4^

Since 2015, various β-lactamase inhibitor combination agents (BLICs) active against CRE isolates were introduced to practice, including ceftazidime/avibactam, meropenem/vaborbactam, and imipenem/relebactam.^1,5^ These agents, along with cefiderocol, represented a significant improvement in the treatment of CRE infections, especially those caused by KPC-producing isolates, which are the main targets of BLICs. However, meropenem/vaborbactam and imipenem/relebactam have limited activity against OXA-48-like producers and none of these newer BLIC agents are active against MBLs, including the NDM, the frequency of which has increased in the last years in some regions.^5,6^ Cefiderocol has shown good activity against serine carbapenemase producers but also has limited activity against some MBLs, especially NDM-5.^7,8^

Aztreonam/avibactam was approved by the European Medicines Agency (EMA) in the European Union in April 2024 (https://www.ema.europa.eu/en/news/new-antibiotic-fight-infections-caused-multidrug-resistant-bacteria; accessed on 23 January 2025). There are many studies demonstrating the potent *in vitro* activity of aztreonam/avibactam against CREs producing all types of clinically relevant carbapenemases and against non–carbapenemase-producing CREs as well.^6,9,10^ In this investigation, we evaluated the activity of aztreonam/avibactam against a large collection of Enterobacterales collected in 46 European medical centres during the 5 years prior to its approval in 2024 for clinical use in Europe to provide a benchmark for future evaluations of the emergence of antimicrobial resistance.

## Materials and methods

### Organism collection

Bacterial isolates were collected via a network of medical sites participating in the SENTRY Antimicrobial Surveillance Program and sent to Element Iowa City (JMI Laboratories; North Liberty, Iowa, USA). Participating centres were requested to gather consecutive bacterial isolates from individual patients hospitalised with the following infection types: pneumonia, bloodstream infection, urinary tract infection, skin and skin structure infection, and intra-abdominal infection. Only isolates established to be the cause of infection were included in the programme. The number of isolates by type of infection as well as the period of the year the isolates were collected varied slightly by geography.

A total of 30 074 Enterobacterales isolates were collected from European medical centres in 2019–2023. Because the epidemiology of antimicrobial resistance varies markedly between Eastern and Western European countries, the results were stratified by geographic region. The Eastern Europe and Mediterranean region (E-EU) included 8074 isolates from 19 medical centres located in 11 countries [Belarus (1 centre, 2019 only), Czech Republic (1 centre), Greece (1 centre), Hungary (3 centres), Israel (2 centres), Poland (1 centre), Romania (2 centres), Russia (3 centres, 2019 only), Slovakia (1 centre), Slovenia 2 centres, and Turkey (2 centres)], while Western Europe (W-EU) included 22 000 isolates from 27 medical centres located in 10 countries Belgium [1 centre], France [4], Germany [7], Ireland [1], Italy [4], Portugal [1], Spain [(3), Sweden (2), Switzerland (1), and the United Kingdom (3)].

### Susceptibility testing

Susceptibility testing was performed by the broth microdilution method described by CLSI standards.^11^ Imipenem/relebactam and cefiderocol were not tested against isolates collected in 2019. All tests were conducted in a central laboratory [Element Iowa City (JMI Laboratories)]. EUCAST breakpoint criteria was applied for aztreonam/avibactam (susceptible at ≤4 mg/L and resistant at >4 mg/L) and comparator agents when available.^12^ Enterobacterales isolates with imipenem or meropenem MIC ≥4 mg/L were categorized as CRE. Imipenem was not employed to indole-positive Proteeae or *Proteus mirabilis* because these organisms show intrinsically elevated imipenem MIC values. Overall, 1105 (3.7%) CRE isolates were identified for further molecular evaluation. Difficult-to-treat (DTR) was defined as a CRE isolate not susceptible to fluoroquinolones (levofloxacin) and aminoglycosides (gentamicin and amikacin) according to EUCAST criteria.^12,13^ Concurrent quality control (QC) testing was performed to ensure proper test conditions and procedures.

### β-Lactamase screening and molecular characterization of CRE isolates with decreased susceptibility to aztreonam/avibactam

All 22 isolates with decreased susceptibility to aztreonam/avibactam (MIC >4 mg/L) were genome sequenced for *in silico* screening of genetic mechanisms potentially contributing to this phenotype. In brief, total genomic DNA was extracted using the ThermoScientific™ KingFisher™ Flex Magnetic Particle Processor (Thermo Fisher Scientific; Cleveland, OH, USA). Sequencing libraries were prepared from total genomic DNA using the Nextera DNA Flex™ or Illumina DNA Prep™ kits (Illumina; San Diego, CA, USA) and sequenced on a Miseq or Nextseq 1000/2000 instrument (Illumina). FASTQ format files for each sample set were assembled independently using the *de novo* assembler SPAdes 3.15.3 with K-values of 21, 33, 55, 77, and 99 plus careful mode on to reduce the number of mismatches.^14^ An in-house proprietary bioinformatic pipeline and a JMI Laboratories-curated resistance gene database (Version 3; uses Python v2.7.9, SPAdes v3.15.3, and BBMap v36.x) based on the NCBI Bacterial Antimicrobial Resistance Reference Gene Database (https://www.ncbi.nlm.nih.gov/bioproject/PRJNA313047) was used for the *in silico* analysis of acquired resistance genes. Genes encoding intrinsic resistance factors (e.g. amino acid alterations in OmpC, OmpF, and PBP3) were assessed relative to a susceptible reference strain.^15^

Expression of the intrinsic *ampC* gene in *Enterobacter cloacae* species complex and *K. aerogenes* isolates was assessed. Isolates were inoculated into fresh cation adjusted Mueller-Hinton broth to 0.5 McFarland units (Thermo Fisher Scientific; Waltham, MA, USA), diluted 1:10 in fresh tryptic soy broth, and grown to log phase (OD_600_ 0.3–0.5). Cells were then mixed 1:2 with RNA Protect (Qiagen; Hilden, Germany), and RNA extraction was performed using the RNA Easy Mini kit (Qiagen) on a Qiacube workstation (Qiagen). Total RNA was treated with RQ1 RNase-free DNase (Promega; Madison, WI, USA) and cleaned using the RNA Easy Mini kit. Gene expression was determined in triplicate for each sample with 0.5 ng of RNA per reaction using the Power SYBR Green RNA-to-C_T_ 1-step kit on a StepOnePlus qPCR workstation (Applied Biosystems-Thermo Fisher Scientific). Gene expression was normalized to expression of *rpsL* and compared to a susceptible control strain (*E. cloacae* species complex, ATCC 700323; *K. aerogenes,* in-house reference strain 29072) using the StepOnePlus analysis platform (Applied Biosystems).

## Results

The antimicrobial susceptibility of the Enterobacterales collection stratified by European region (E-EU and W-EU) is shown in Table 1. Aztreonam/avibactam was active against 99.8% and >99.9% of Enterobacterales from E-EU and W-EU, respectively. Susceptibility rates for comparator agents were generally lower in E-EU compared to W-EU. Ceftazidime/avibactam and meropenem/vaborbactam retained activity against 94.9%–96.3% of Enterobacterales from E-EU and 99.6%–99.7% of Enterobacterales from W-EU. Cefiderocol was active against 97.4%/99.2% of isolates from E-EU/W-EU.

**Table 1.** Antimicrobial susceptibility of Enterobacterales and resistant subsets from European medical centres stratified by Eastern Europe (E-EU) and Western Europe (W-EU)

	E-EU				W-EU			
Antimicrobial/organism	MIC_50_	MIC_90_	%S^a^	%R^a^	MIC_50_	MIC_90_	%S^a^	%R^a^
**Enterobacterales (no.)**	**(8074)**				**(22 000)**			
Aztreonam/avibactam	≤0.03	0.25	99.8	0.2	≤0.03	0.12	>99.9	<0.1
Ceftazidime/avibactam	0.12	1	96.3	3.7	0.12	0.25	99.6	0.4
Ceftolozane/tazobactam	0.25	>16	83.6	16.4	0.25	1	93.8	6.2
Meropenem/vaborbactam	0.03	0.12	94.9	4.7	0.03	0.06	99.7	0.4
Imipenem/relebactam	0.12	2	94.3^b^	6.2	0.12	1	98.4^b^	1.7
Piperacillin/tazobactam	2	>128	73.6	26.4	2	32	85.3	14.7
Cefiderocol	2	4	97.4	30.6	1	4	99.2	18.5
Ceftriaxone	0.12	>8	62.6^c^	36.6	≤0.06	>8	80.1^c^	18.8
Meropenem	0.03	1	91.1^c^	7.3	0.03	0.06	98.6^c^	1.0
Levofloxacin	0.12	32	63.2	32.0	0.06	8	82.2	14.9
Gentamicin	0.5	>16	81.1^d^	18.9	0.5	2	91.4^d^	8.6
Amikacin	2	8	90.6^d^	9.4	2	4	98.1^d^	1.9
Tigecycline	0.25	2			0.25	1		
Colistin	0.25	>8	81.6	18.4	0.25	>8	82.9	17.1
**CRE (no.)**	**(757)**				**(347)**			
Aztreonam/avibactam	0.25	0.5	99.6	0.5	0.25	0.5	99.7	0.3
Ceftazidime/avibactam	2	>32	63.0	37.0	2	>32	76.4	23.6
Meropenem/vaborbactam	16	>32	45.1	55.4	1	>32	80.3	22.5
Imipenem/relebactam	8	>16	46.9^b^	53.1	0.5	16	68.3^b^	33.1
Cefiderocol	1	4	74.8	21.9	1	4	87.6	18.5
Gentamicin	>16	>16	38.4^d^	61.6	2	>16	51.7^d^	48.3
Amikacin	32	>32	35.7^d^	64.3	4	>32	65.4^d^	34.6
Levofloxacin	32	>32	6.3	88.0	16	>32	14.2	82.7
Tigecycline	1	2			0.5	2		
Colistin	0.25	>8	66.1	33.9	0.25	>8	88.4	11.6
**DTR (no.)**	**(344)**				**(62)**			
Aztreonam/avibactam	0.25	0.5	100.0	0.0	0.25	0.5	100.0	0.0
Ceftazidime/avibactam	2	>32	55.5	44.6	2	>32	66.1	33.9
Meropenem/vaborbactam	32	>32	28.5	71.8	2	>32	61.3	37.9
Imipenem/relebactam	16	>16	34.9^b^	65.1	0.25	>16	60.3^b^	38.7
Cefiderocol	2	4	67.3	32.7	1	4	86.8	13.2
Gentamicin	>16	>16	0.0^d^	100.0	>16	>16	0.0^d^	100.0
Amikacin	>32	>32	0.0^d^	100.0	>32	>32	0.0^d^	100.0
Levofloxacin	32	>32	0.0	94.8	16	>32	0.0	100.0
Tigecycline	1	2			0.5	2		
Colistin	0.25	>8	58.4	41.6	0.25	>8	74.2	25.8
**Aztreonam/avibactam-NS (no.)**	**(15)**				**(7)**			
Aztreonam/avibactam	8	>16	0.0	100.0	8		0.0	100.0
Ceftazidime/avibactam	4	>32	86.7	13.3	4		71.4	28.6
Ceftolozane/tazobactam	>16	>16	0.0	100.0	>16		0.0	100.0
Meropenem/vaborbactam	0.06	32	86.7	13.3	0.06		100.0	0.0
Imipenem/relebactam	0.25		88.9^b^	11.1	0.25		100.0^b^	0.0
Piperacillin/tazobactam	>128	>128	0.0	100.0	128		0.0	100.0
Aztreonam	>16	>16	0.0	100.0	>16		0.0	100.0
Cefiderocol	1	2	93.3	6.7	1		85.7	14.3
Ceftriaxone	>8	>8	0.0^c^	100.0	>8		0.0^c^	100.0
Meropenem	0.06	32	86.7^c^	13.3	0.12		100.0^c^	0.0
Levofloxacin	16	>32	0.0	100.0	1		42.9	42.9
Gentamicin	1	>16	53.3^d^	46.7	0.5		100.0^d^	0.0
Amikacin	4	8	93.3^d^	6.7	4		71.4^d^	28.6
Tigecycline	0.25	1			0.25			
Colistin	0.12	0.25	100.0	0.0	0.12		100.0	0.0

CRE, carbapenem-resistant Enterobacterales; DTR, difficult-to-treat defined as a CRE isolate not susceptible to fluoroquinolones (levofloxacin) and aminoglycosides (gentamicin and amikacin) per EUCAST criteria; E-EU, Eastern Europe and Mediterranean region; W-EU, Western Europe; NS, nonsusceptible.

^a^Criteria as published by EUCAST (2025).

^b^All Enterobacterales species were included in the analysis, but EUCAST exclude organisms from the family Morganellaceae.

^c^Using non-meningitis breakpoints.

^d^For infections originating from the urinary tract. For systemic infections, aminoglycosides must be used in combination with other active therapy.

Only aztreonam/avibactam showed good activity (>90% susceptibility) against CRE isolates, with MIC_50/90_ values of 0.25/0.5 mg/L in both European regions and susceptibility rates of 99.6% in E-EU and 99.7% in W-EU (Table 1). Cefiderocol was the second most active compound against CREs, with susceptibility rates of 74.8% in E-EU and 87.6% in W-EU. Ceftazidime/avibactam, meropenem/vaborbactam, and imipenem/relebactam were active against 68.3%–80.3% of CRE isolates from W-EU, and 45.1%–63.0% against CRE isolates from E-EU (susceptibility; Table 1).

All DTR (CRE nonsusceptible to fluoroquinolones and aminoglycosides) isolates from both E-EU and W-EU were susceptible to aztreonam/avibactam (MIC_50/90_, 0.25/0.5 mg/L in both regions; Table 1). Cefiderocol retained moderate activity against DTR isolates with susceptibility rates of 67.3% in E-EU and 86.8% in W-EU; whereas ceftazidime/avibactam, meropenem/vaborbactam, and imipenem/relebactam showed limited activity against these organisms in both E-EU (28.5%–55.5% susceptibility) in W-EU (60.3%–66.1% susceptible; Table 1).

Notably, 86.7%/100.0% of aztreonam/avibactam-nonsusceptible isolates from E-EU/W-EU (90.9% overall) remained susceptible to meropenem per EUCAST criteria (Table 1).

Table 2 displays CRE rates, carbapenemase distributions among CREs, and susceptibility of CRE isolates to aztreonam/avibactam, ceftazidime/avibactam, meropenem/vaborbactam, and cefiderocol stratified by European region and country. Overall CRE frequency was markedly higher in E-EU (9.4%) compared W-EU (1.6%), with great variation among countries in both regions. Among E-EU countries, CRE frequency varied from <1% in Czech Republic, Hungary, and Slovenia to >16% in Greece (16.8%), Poland (18.0%), and Russia (17.8%); whereas in W-EU, the CRE frequency was <1% in 7 of 10 countries surveyed, and the highest frequencies were observed in Italy (5.0%), Portugal (2.0%), and Spain (1.8%).

**Table 2.** Results of carbapenem-resistant Enterobacterales (CRE) characterization stratified by country

European region/	No. of	No. of	% of	% of CRE with:				% of CRE susceptible^a^ to:			
Country	isolates	CRE	CRE	KPC	MBL	OXA-48	No Cbase	Cefiderocol	MEM/VAB	CAZ/AVI	ATM/AVI
**E-EU**											
Belarus	103	25	24.3	0.0	72.0	32.0	0.0	22.2	16.0	28.0	100.0
Czech Republic	538	4	0.7	75.0	25.0	0.0	0.0	50.0	75.0	75.0	100.0
Greece	1035	174	16.8	55.7	43.1	0.6	0.6	70.3	65.5	56.9	100.0
Hungary	777	5	0.6	0.0	40.0	0.0	60.0	100.0	20.0	20.0	100.0
Israel	910	25	2.7	28.0	40.0	0.0	32.0	78.3	68.0	56.0	100.0
Poland	728	131	18.0	3.8	38.9	9.7	52.7	76.0	54.6	59.5	98.5
Romania	406	34	8.4	23.5	58.8	17.6	0.0	53.3	41.2	41.2	100.0
Russia	343	61	17.8	29.5	18.0	51.7	1.6	NT	54.1	82.0	100.0
Slovakia	214	27	12.6	48.1	0.0	0.0	51.9	100.0	100.0	100.0	100.0
Slovenia	1099	1	0.1	0.0	100.0	0.0	0.0	100.0	0.0	0.0	100.0
Turkey	1921	270	14.1	10.0	31.9	57.4	1.9	75.4	21.1	68.1	99.6
E-EU Total	8074	757	9.4	23.9 ^b^	36.5	26.8^b^	13.3	74.8	45.1	63.0	99.6
**W-EU**											
Belgium	981	6	0.6	0.0	50.0	40.0	16.7	80.0	50.0	50.0	100.0
France	2909	22	0.8	4.5	72.7	10.5	13.6	63.2	31.8	27.3	95.5
Germany	4885	38	0.8	10.5	36.8	41.9	18.4	77.4	57.9	65.8	100.0
Ireland	995	3	0.3	0.0	66.7	33.3	0.0	66.7	66.7	33.3	100.0
Italy	3870	194	5.0	79.4	18.6	0.5	1.5	90.7	86.1	80.9	100.0
Portugal	968	19	2.0	94.7	0.0	0.0	5.3	100.0	100.0	100.0	100.0
Spain	3280	57	1.7	45.6	17.5	32.1	7.0	97.4	93.0	82.5	100.0
Sweden	1076	1	0.1	0.0	0.0	0.0	100.0	NT	100.0	100.0	100.0
Switzerland	930		0.0								
UK	2106	7	0.3	71.4	28.6	0.0	0.0	83.3	71.4	85.7	100.0
W-EU Total	22 000	347	1.6	60.2^b^	23.9	10.4^b^	5.8	87.6	80.4	76.4	99.7

ATM-AVI, aztreonam/avibactam; CAZ/AVI, ceftazidime-avibactam; Cbase, carbapenemase; CRE, carbapenem-resistant Enterobacterales; KPC, *Klebsiella pneumoniae* carbapenemase; MBL, metallo-β-lactamase; MEM/VAB, meropenem/vaborbactam; OXA, oxacillinase.

^a^Based on EUCAST criteria (2025).

^b^Exclude isolates that co-produce an MBL but include isolates that produces a KPC and an OXA-48-like (3 isolates in E-EU and 1 isolate in W-EU).

MBL was the predominant carbapenemase type among CREs from E-EU (Table 2). An MBL was identified in 36.5% of CREs from E-EU, and it was the most common carbapenemase type in Belarus (72.0% of CREs), Hungary (40.0%), Israel (40.0%), Poland (38.9%), Romania (58.8%), and Slovenia (100.0%, only 1 CRE identified). OXA-48-like was the second most common carbapenemase type in E-EU, accounting for 26.8% of CREs. OXA-48-like was the most common carbapenemase type found in Russia (51.7% of CREs) and Turkey (57.4%). KPC was identified in only 23.5% of E-EU CREs and predominated only in Czech Republic [75.0% (3/4) of CREs], Greece (55.7%), and Slovakia (48.1%). Notably, a carbapenemase gene was not identified on the majority of CRE isolates from Hungary (60.0% of CREs), Poland (52.7%), and Slovakia (51.7%; Table 2).

The distributions of carbapenemases among CRE isolates were clearly different in W-EU compared to E-EU. KPC was the predominant carbapenemase type in W-EU countries (59.9% of CREs), followed by MBL (23.9%), and OXA-48-like (11.0%; Table 2). KPC predominated in Italy (79.4% of CREs), Portugal (94.7%), Spain (71.4%), and UK (59.9%), whereas MBL predominated in Belgium (50.0%), France (72.7%), and Ireland (66.7%). OXA-48-like predominated only in Germany (41.9% of CREs; Table 2).

Aztreonam/avibactam retained activity against 100.0% of CRE isolates in all countries except Poland (98.5% susceptible), Turkey (99.6%), and France (95.5%; Table 2). The activities of ceftazidime/avibactam and meropenem/vaborbactam varied widely based on the frequency of each carbapenemase type in each country but were generally limited in most countries in both regions (Table 2).

The activities of aztreonam/avibactam and comparator agents against CRE isolates stratified by carbapenemase type and European region are shown in Table 3. Aztreonam/avibactam was active against all (100.0%) of carbapenemase-producing CREs and 95.0% of non–carbapenemase-producing CREs from W-EU. When tested against CRE isolates from E-EU, aztreonam/avibactam was active against 99.8% (655/656) of carbapenemase producers and 98.0% (99/101) of non-carbapenemase producers. Cefiderocol retained good activity against KPC producers (92.5% susceptible) and OXA-48-like producers (91.7% susceptible), but only 63.5% of MBL producers were susceptible to cefiderocol, including 59.4% of E-EU and 77.1% of W-EU isolates (Table 3). Ceftazidime/avibactam was highly active against KPC (99.5% susceptible) and OXA-48-like (100.0%) producers but was inactive against MBL producers. Meropenem/vaborbactam and imipenem/relebactam were highly active against KPC producers (99.0% and 99.7% susceptible, respectively), but both agents showed very limited activity against MBL and OXA-48-like producers. The most active agents against the non–carbapenemase-producing CRE isolates collection (*n* = 121) was aztreonam/avibactam (97.5% susceptibility), followed by imipenem/relebactam (96.7% susceptible), ceftazidime/avibactam (95.9% susceptible), meropenem/vaborbactam (95.8% susceptible), and cefiderocol (88.9% susceptible; Table 3).

**Table 3.** Antimicrobial susceptibility of CRE isolates stratified by carbapenemase type and European region

European region/antimicrobial agent	% susceptible^a^ (no. of isolates)				
	All CRE	KPC producers	MBL producers	OXA-48 producers	No carbapenemase identified
**E-EU**	**(757)**	**(177)**	**(276)**	**(203)**	**(101)**
Aztreonam/avibactam	99.6	100.0	100.0	99.5	98.0
Ceftazidime/avibactam	63.0	100.0	0.4	100.0	95.0
Meropenem/vaborbactam	45.1	99.3	10.5	20.2	95.0
Imipenem/relebactam^b^	46.9	100.0	1.1	31.2	95.9
Cefiderocol	74.8	91.9	59.4	90.7	86.9
Amikacin^c^	38.4	37.3	23.6	31.5	74.3
Gentamicin^c^	35.7	54.2	30.1	34.5	41.6
Levofloxacin	6.3	3.4	6.5	7.4	8.9
Colistin	66.1	68.9	70.9	54.2	72.3
**W-EU**	**(347)**	**(208)**	**(83)**	**(36)**	**(20)**
Aztreonam/avibactam	99.7	100.0	100.0	100.0	95.0
Ceftazidime/avibactam	76.4	99.0	3.6	100.0	100.0
Meropenem/vaborbactam	80.4	98.2	36.1	66.7	100.0
Imipenem/relebactam^b^	68.3^b^	97.0	7.7	40.6	100.0
Cefiderocol	87.6	93.2	77.1	95.8	100.0
Amikacin^c^	51.7	63.5	62.7	86.1	60.0
Gentamicin^c^	65.4	52.2	43.4	63.9	60.0
Levofloxacin	14.2	9.2	20.5	19.4	30.0
Colistin	88.4	89.9	83.1	88.9	94.7
**Total (697)**	**(1104)**	**(385)**	**(359)**	**(239)**	**(121)**
Aztreonam/avibactam	99.6	100.0	100.0	99.6	97.5
Ceftazidime/avibactam	67.2	99.5	1.1	100.0	95.9
Meropenem/vaborbactam	56.2	99.0	16.4	27.2	95.8
Imipenem/relebactam^b^	54.2	99.7	2.1	33.1	96.7
Cefiderocol	84.2	92.5	63.5	91.7	88.9
Amikacin^c^	45.1	51.4	32.6	39.7	71.9
Gentamicin^c^	42.7	53.1	33.1	38.9	44.6
Levofloxacin	8.9	6.5	9.8	9.2	12.4
Colistin	73.1	80.2	73.7	59.4	75.8

CRE, carbapenem-resistant Enterobacterales; E-EU, Eastern Europe and Mediterranean region; KPC, *Klebsiella pneumoniae* carbapenemase; MBL, metallo-β-lactamase; OXA, oxacillinase; W-EU, Western Europe.

^a^EUCAST criteria (2025).

^b^All Enterobacterales species were included in the analysis, but CLSI and EUCAST exclude organisms from the family Morganellaceae.

^c^For infections originating from the urinary tract. For systemic infections, aminoglycosides must be used in combination with other active therapy.

**Figure 1.**
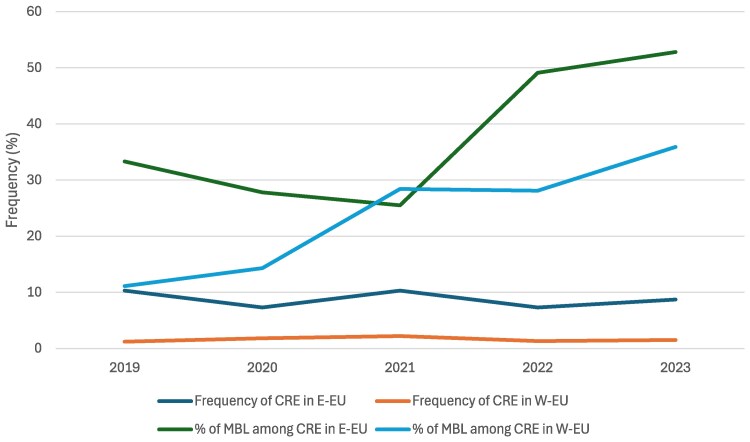
Yearly analysis of the frequencies of carbapenem-resistant Enterobacterales (CRE) and the proportion of metallo-β-lactamases (MBLs) among CREs.

Yearly analysis of susceptibility to selected β-lactams showed slight variation of susceptibility rates without a clear trend of increase or decrease over the years of the investigation (Table 4). The frequency of CRE also remained relatively stable during the study period; however, the proportion of MBL producers among CRE isolates increased markedly in the last 2 years of the investigation (Figure 1). In E-EU, the percentage of MBL producers among CREs showed an initial decreased from 33.3% in 2019% to 25.5% in 2021 and then increased to 49.1% in 2022% and 52.8% in 2023. In W-EU, the proportion of MBL producers among CREs increased from 11.1% in 2019% to 35.9% in 2023 (Figure 1).

**Table 4.** Yearly susceptibility rates for selected β-lactam compounds.

	% Susceptible per EUCAST (no. of isolates)				
	2019	2020	2021	2022	2023
Eastern Europe^a^					
Aztreonam-avibactam	99.5	99.7	99.9	99.8	99.9
Ceftazidime-avibactam	96.5	97.4	97.1	95.2	96.4
Ceftriaxone	59.9	63.9	62.6	66.8	67.5
Western Europe					
Aztreonam-avibactam	>99.9	>99.9	>99.9	>99.9	>99.9
Ceftazidime-avibactam	99.8	99.7	99.3	99.7	99.4
Ceftriaxone	80.9	79.1	78.6	82.3	79.7

^a^Exclude isolates from Belarus, Russia, and Slovakia because these countries did not participate during the entire period of the study.

A summary of the molecular characterization results for the 22 aztreonam/avibactam–nonsusceptible isolates (MIC >4 mg/L) is displayed in Table 5 and a full genotypic description of these isolates can be found in Table S1 (available as Supplementary data at *JAC* Online). The collection includes 16 *Escherichia coli*, 5 *Enterobacter cloacae* species complex (4 *E. hormaechei* and 1 *E. asburiae*), and 1 *Klebsiella aerogenes*. Most *E. coli* isolates were from Turkey (12/16) or Italy (3/16). The *E. cloacae* species complex isolates (*n* = 5) were from France (2), Poland (2), and Italy (1), and the *K. aerogenes* was from Italy. E-EU contributed with 26.8% of Enterobacterales for this investigation and had 68.2% (15/22) aztreonam/avibactam-nonsusceptible isolates.

**Table 5.** Molecular characterization of aztreonam-avibactam–nonsusceptible Enterobacterales from European medical centres (2019–2023)

Collection	Organism	Year	Country	MLST	MIC (mg/L)				AmpC/	PBP	OmpC	OmpF	AmpC
Number					ATM-AVI	CAZ-AVI	MEM-VAB	FDR	ESBL	alteration			expression^a^
1301084	*E. coli*	2023	Israel	361	8	4	0.03	1	CMY-42	P333insYRIN	^b^	^b^	Not done
1130864	*E. coli*	2019	Italy	405	8	8	0.03	2	CTX-M-15	P333insYRIK	^b^	^b^	Not done
1155924	*E. coli*	2020	Italy	6870	16	4	0.06	0.12	CMY-145, OXA-1	P333insYRIK	^b^	^b^	Not done
1211023	*E. coli*	2021	Italy	405	8	4	0.03	1	CTX-M-15, OXA-1	P333insYRIK	^b^	^b^	Not done
1183154	*E. coli*	2020	Poland	410	16.1	32.1	32	0.5	CMY-141, TEM-190	P333insYRIK	^b^	^b^	Not done
1108470	*E. coli*	2019	Turkey	410	8	2	0.03	1	CMY-42, CTX-M-15, OXA-1	P333insYRIK	^b^	^b^	Not done
1108523	*E. coli*	2019	Turkey	410	8	2	0.03	0.5	CMY-42, OXA-1	P333insYRIK	^b^	^b^	Not done
1108694	*E. coli*	2019	Turkey	410	8	2	0.06	0.5	CMY-141	P333insYRIK	^b^	^b^	Not done
1114251	*E. coli*	2019	Turkey	410	16	8	0.06	1	CMY-42, CTX-M-15, OXA-1	P333insYRIK	^b^	^b^	Not done
1114255	*E. coli*	2019	Turkey	410	8	8	0.5	1	CMY-42, OXA-48, CTX-M-14	P333insYRIK	^b^	^b^	Not done
1174299	*E. coli*	2020	Turkey	410	8	2	0.03	0.5	CMY-42	P333insYRIK	^b^	^b^	Not done
1177727	*E. coli*	2020	Turkey	4450	16	4	0.5	0.5	CMY-42, OXA-244, CTX-M-15	P333insYRIK	^b^	^b^	Not done
1225386	*E. coli*	2021	Turkey	648	8	2	0.5	0.25	CMY-42, OXA-244	P333insYRIK	^b^	^b^	Not done
1262738	*E. coli*	2022	Turkey	410	8	2	0.03	1	CMY-141, CTX-M-15, OXA-1	P333insYRIK	^b^	^b^	Not done
1262754	*E. coli*	2022	Turkey	410	8	4	0.25	2	CMY-42, CTX-M-15, OXA-181, OXA-1,	P333insYRIN	^b^	^b^	Not done
1269603	*E. coli*	2022	Turkey	410	16	4	0.03	2	CMY-141, CTX-M-3, OXA-1	P333insYRIK	^b^	^b^	Not done
1257664	*E. asburiae*	2022	France	24	8	2	0.25	2	−^c^	I20 V,V48I, V545I	Q66X	T183A,L234 V,G303N	1.9
1254173	*E. hormaechei*	2022	France	1424-like	8	4	0.06	16	−^c^	P311S	E188D	WT	1664.3
1279134	*E. hormaechei*	2023	Italy	50-like	16	>32	0.06	0.25	−^c^	WT	M1X	L60fs_G64X	227.5
1118254	*E. hormaechei*	2019	Poland	78	16	2	0.06	8	SHV-12	V20I, E258_ S259insE	K224E,N337G	L211I	596.6
1183311	*E. hormaechei*	2020	Poland	89	>16	>32	32	1	CTX-M-15, OXA-1	WT	E188D,L211I,Y229F,L360P	G223_G223del,K224M,D226X	86.6
1301571	*K. aerogenes*	2023	Italy	168	8	>32	0.03	1	−^c^	A317 V,A498T	F230Y,V231F,T234S,G236Q,G237R,R238T	F168L, W315X	246.8

ATM-AVI, aztreonam/avibactam; CAZ/AVI, ceftazidime-avibactam; FDR, cefiderocol; MLST, multi-locus sequence typing; WT, wild type.

^a^mRNA expression compared to a control strain.

^b^See Supplemental Table S1 for full genotype description.

^c^An acquired β-lactamase gene was not detected.

All aztreonam/avibactam-nonsusceptible *E. coli* showed amino acid alterations in the PBP3, either as an ‘YRIK’ or ‘YRIN’ insertion after amino acid 333. Moreover, most *E. coli* isolates carried a CMY β-lactamase (either CMY-42, CMY-141, or CMY-145), usually with a CTX-M and/or an OXA type (Table 4). Five STs were observed among these 16 *E. coli* isolates, with 10 isolates from one site in Turkey belonging to ST410. Notably, these 10 isolates showed different β-lactamase profiles. High-level expression of *ampC* and amino acid alterations within OmpC and/or OmpF (i.e. stop codons) were found among the *E. cloacae* species complex and *K. aerogenes* isolates (Table 5 and Table S1).

## Discussion

We evaluated the *in vitro* activity of aztreonam/avibactam and key comparator agents against a large collection of Enterobacterales (*n* = 30 074) collected from 46 European medical centres in the 5-year period prior to its approval for clinical use in Europe. The results of this investigation provide a valuable benchmark for monitoring the development of resistance to this compound once it becomes widely used in Europe. Aztreonam/avibactam exhibited potent *in vitro* activity and almost complete coverage against Enterobacterales causing infections in the European medical centres evaluated, and our results corroborate other recent publications. Wise *et al.*^10^ reported aztreonam/avibactam results tested against 24 937 Enterobacterales collected from 27 countries worldwide (excluding US) in 2019–2021, and aztreonam/avibactam inhibited 99.8% of isolates at ≤8 mg/L, including 99.5% of MDR, 98.7% of XDR, and 99.1% of CRE. Rosseline *et al.*^9^ tested 18 713 Enterobacterales collected from 232 medical centres worldwide in 2019. Aztreonam/avibactam inhibited 99.9% of isolates at ≤8 mg/L and exhibited potent activity (>99.0%; MIC_90,_ 0.5 mg/L) against isolates producing MBLs, KPC, and/or OXA-48.

Although a few studies have demonstrated that aztreonam/avibactam has near complete activity against Enterobacterales,^6,9,10^ resistance may emerge once the compound is widely used in the clinical setting. In the present study, we observed only 22 isolates with decreased susceptibility (MIC >4 mg/L) to aztreonam/avibactam, which represented 0.07% of Enterobacterales isolates tested. Notably, 90.9% (20/22) were meropenem-susceptible per EUCAST criteria (MIC ≤2 mg/L) and only 4 isolates (18.2%) were categorized as CRE (MIC >4 mg/L for imipenem and/or meropenem). Moreover, the main mechanisms affecting aztreonam/avibactam *in vitro* activity reported here (ESBL and/or plasmid AmpC with PBP3 mutations) have been reported by other investigators and may have been triggered by the clinical use of aztreonam plus ceftazidime/avibactam or other anti-CRE agents, such as cefiderocol or meropenem/vaborbactam.^16–19^

Among 22 aztreonam/avibactam-nonsusceptible isolates identified in the present study, only 2 were resistant to meropenem/vaborbactam or cefiderocol (MIC >4 mg/L), and 4 were resistant to ceftazidime/avibactam. Notably, MBL or KPC were not identified in any of the aztreonam/avibactam-nonsusceptible isolates.

*Escherichia coli* represented 72.7% (16/22) of the aztreonam/avibactam-nonsusceptible Enterobacterales identified in the present study. As reported by other investigators, aztreonam/avibactam resistance in *E. coli* was mainly related to amino acid alterations in the PBP3 sequence plus the production of a CMY β-lactamase and/or an ESBL.^17,20–22^ Various agents, such as piperacillin, aztreonam, ceftazidime, ceftriaxone, cefotaxime, cefepime, and cefiderocol preferably bind to PBP3, an essential transpeptidase responsible for *E. coli* cell division. Therefore, insertion mutations in PBP3 adversely affect the activity of these agents.^16,23^ Remarkably, the emergence of *E. coli* isolates with ‘YRIK’ or ‘YRIN’ insertions in PBP3 predates the clinical use of avibactam or cefiderocol and appears to be related to some high-risk clones, such as MLSTs 410, 167, and 405.^22^

The remaining aztreonam/avibactam nonsusceptible isolates were *E. cloacae* species complex (5 isolates) and *Klebsiella aerogenes* (former *Enterobacter aerogenes*; 1 isolate). In contrast to *E. coli*, scarce data are available on mechanisms of resistance to aztreonam/avibactam in *Enterobacter* spp. and *Klebsiella* spp. other than *K. pneumoniae.*^18^ Interestingly, 4 of 5 *E. cloacae* complex isolates were identified as *E. hormaechei* by whole genome sequencing; all 4 isolates had unique MLST. Since *E. cloacae* complex isolates are not usually identified to the species level, scarce information is available about the epidemiology of this organism. Moreover, *E. hormaechei* appears to be the dominant species among carbapenem-resistant *E. cloacae* complex.^24–26^

Interestingly, we did not detect any *K. pneumoniae* with decreased susceptibility to aztreonam/avibactam among more than 6000 isolates evaluated. Although cross-resistance to aztreonam/avibactam and cefiderocol among ceftazidime/avibactam-resistant *K. pneumoniae* producing KPC-3 mutants has been reported,^16,19^ we did not identify any *K. pneumoniae* showing this resistance phenotype in this investigation.

The surveillance data we present in this report have limitations. The number of medical centres in some countries were very limited, especially in E-EU, and not all medical centres participated in the programme during the entire 5-year period of the investigation. For instance, medical centres from Belarus and Russia contributed isolates only in 2019. As participating centres leave the programme, additional centres from that region are added, with the goal of maintaining a robust and broadly representative sample from as many countries and regions as possible.

In summary, the surveillance data present here provides a valuable baseline for monitoring the *in vitro* activity of aztreonam/avibactam. This surveillance programme will continue to monitor the *in vitro* activity of aztreonam/avibactam in European medical centres following its approval in EU. Our results also highlight the importance of comprehensive surveillance programmes to monitor the emergence of resistance mechanisms to newly approved antimicrobial agents, especially in cases where resistant determinants seem to be associated with some high-risk clonal types (*E. coli* ST410, 167, and 405). Molecular characterization of resistance subsets is essential for better understanding the epidemiology of these isolates and resistance markers, and for guiding the implementation of infection control measures and antimicrobial therapy guidelines.

## Supplementary Material

dkaf161_Supplementary_Data

## Transparency declarations

None of the authors has a conflict of interest. H.S.S., J.H.K., M.L.W., M.C., and R.E.M. are employees of Element Iowa City (JMI Laboratories), which was paid consultant to Pfizer in connection with the development of manuscript. Element Iowa City (JMI Laboratories) was contracted to perform services in 2022–2024 for AbbVie, AimMax Therapeutics, Amicrobe, Inc., Appili Therapeutics, Armata Pharmaceuticals, Astellas Pharma, Inc., Basilea Pharmaceutica AG, Biosergen AB, Bugworks, Cerba Research NV, Cidara Therapeutics, Cipla USA Inc., ContraFect Corporation, CorMedix Inc., Crestone, Inc., Curza Global, LLC, Diamond V, Discuva Ltd., Entasis Therapeutics, Enveda Biosciences, Evopoint Biosciences, Fedora Pharmaceuticals, Fox Chase Chemical Diversity Center, Genentech, Gilead Sciences, Inc., GSK plc, Iterum Therapeutics plc, Janssen Biopharma, Johnson & Johnson, Kaleido Biosciences, LifeMine Therapeutics, Medpace, Inc, Lysovant Sciences, Inc., Meiji Seika Pharma, Melinta Therapeutics, Menarini Group, Merck & Co., MicuRx Pharmaceutical Inc., Mundipharma International Ltd., Mutabilis, Nabriva Therapeutics, National Cancer Institute, National Institutes of Health, Ohio State University, Omnix Medical Ltd., Paratek Pharmaceuticals, PolyPid Ltd., PPD, Prokaryotics, Inc., Pulmocide Ltd, Qpex Biopharma, Revagenix, Roche Holding AG, Roivant Sciences, Scynexis, Inc., Shionogi & Co., Ltd., Sinovent Pharmaceuticals, Inc., Spero Therapeutics, Sumitovant Biopharma, Inc., TenNor Therapeutics, U.S. Food and Drug Administration, VenatoRx Pharmaceuticals, Washington University, Watershed Medical, LLC, Wockhardt, and Zoetis, Inc.

## Supplementary data

Table S1 is available as Supplementary data at *JAC* Online.

## References

[1] Tamma PD, Heil EL, Justo JA et al Infectious Diseases Society of America 2024 guidance on the treatment of antimicrobial-resistant gram-negative infections. Clin Infect Dis 2024. 10.1093/cid/ciae403 Online ahead of print 39108079

[2] Paniagua-Garcia M, Bravo-Ferrer JM, Perez-Galera S et al Attributable mortality of infections caused by carbapenem-resistant enterobacterales: results from a prospective, multinational case-control-control matched cohorts study (EURECA). Clin Microbiol Infect 2024; 30: 223–30. 10.1016/j.cmi.2023.11.008 38267096

[3] Logan LK, Weinstein RA. The epidemiology of carbapenem-resistant Enterobacteriaceae: the impact and evolution of a global menace. J Infect Dis 2017; 215: S28–36. 10.1093/infdis/jiw282 28375512 PMC5853342

[4] Ma J, Song X, Li M et al Global spread of carbapenem-resistant Enterobacteriaceae: epidemiological features, resistance mechanisms, detection and therapy. Microbiol Res 2023; 266: 127249. 10.1016/j.micres.2022.127249 36356348

[5] Barbier F, Hraiech S, Kerneis S et al Rationale and evidence for the use of new beta-lactam/beta-lactamase inhibitor combinations and cefiderocol in critically ill patients. Ann Intensive Care 2023; 13: 65. 10.1186/s13613-023-01153-6 37462830 PMC10354316

[6] Sader HS, Mendes RE, Carvalhaes CG et al Changing epidemiology of carbapenemases among carbapenem-resistant enterobacterales from United States hospitals and the activity of aztreonam-avibactam against contemporary enterobacterales (2019–2021). Open Forum Infect Dis 2023; 10: ofad046. 10.1093/ofid/ofad046 36846612 PMC9945928

[7] Karakonstantis S, Rousaki M, Kritsotakis EI. Cefiderocol: systematic review of mechanisms of resistance, heteroresistance and in vivo emergence of resistance. Antibiotics (Basel) 2022; 11: 723. 10.3390/antibiotics11060723 35740130 PMC9220290

[8] Kaye KS, Naas T, Pogue JM et al Cefiderocol, a siderophore cephalosporin, as a treatment option for infections caused by carbapenem-resistant enterobacterales. Infect Dis Ther 2023; 12: 777–806. 10.1007/s40121-023-00773-6 36847998 PMC10017908

[9] Rossolini GM, Stone G, Kantecki M et al In vitro activity of aztreonam/avibactam against isolates of enterobacterales collected globally from ATLAS in 2019. J Glob Antimicrob Resist 2022; 30: 214–21. 10.1016/j.jgar.2022.06.018 35760303

[10] Wise MG, Karlowsky JA, Mohamed N et al In vitro activity of aztreonam-avibactam against enterobacterales isolates collected in Latin America, Africa/Middle East, Asia, and eurasia for the ATLAS global surveillance program in 2019–2021. Eur J Clin Microbiol Infect Dis 2023; 42: 1135–43. 10.1007/s10096-023-04645-2 37526796 PMC10427541

[11] CLSI . M07Ed12. Methods for Dilution Antimicrobial Susceptibility Tests for Bacteria That Grow Aerobically. CLSI, 2024.

[12] EUCAST . Breakpoint Tables for Interpretation of MICs and Zone Diameters Version 15.0. European Committee on Antimicrobial Susceptibility Testing, 2025.

[13] Tamma PD, Aitken SL, Bonomo RA et al Infectious Diseases Society of America 2022 guidance on the treatment of extended-spectrum beta-lactamase producing enterobacterales (ESBL-E), carbapenem-resistant enterobacterales (CRE), and Pseudomonas aeruginosa with difficult-to-treat resistance (DTR-P. aeruginosa). Clin Infect Dis 2022; 75: 187–212. 10.1093/cid/ciac268 35439291 PMC9890506

[14] Bankevich A, Nurk S, Antipov D et al SPAdes: a new genome assembly algorithm and its applications to single-cell sequencing. J Comput Biol 2012; 19: 455–77. 10.1089/cmb.2012.0021 22506599 PMC3342519

[15] Mendes RE, Jones RN, Woosley LN et al Application of next-generation sequencing for characterization of surveillance and clinical trial isolates: analysis of the distribution of beta-lactamase resistance genes and lineage background in the United States. Open Forum Infect Dis 2019; 6: S69–78. 10.1093/ofid/ofz004 30895217 PMC6419912

[16] Boattini M, Bianco G, Comini S et al In vivo development of resistance to novel beta-lactam/beta-lactamase inhibitor combinations in KPC-producing Klebsiella pneumoniae infections: a case series. Eur J Clin Microbiol Infect Dis 2024; 43: 2407–17. 10.1007/s10096-024-04958-w 39384682 PMC11608324

[17] Ma K, Zong Z. Resistance to aztreonam-avibactam due to CTX-M-15 in the presence of penicillin-binding protein 3 with extra amino acids in Escherichia coli. Front Microbiol 2022; 13: 1047109. 10.3389/fmicb.2022.1047109 36406430 PMC9674307

[18] Wu S, Ma K, Feng Y et al Resistance to aztreonam-avibactam due to a mutation of SHV-12 in Enterobacter. Ann Clin Microbiol Antimicrob 2023; 22: 49. 10.1186/s12941-023-00605-y 37365592 PMC10294450

[19] Xiang X, Kong J, Zhang J et al Multiple mechanisms mediate aztreonam-avibactam resistance in Klebsiella pneumoniae: driven by KPC-2 and OmpK36 mutations. Int J Antimicrob Agents 2025; 65: 107425. 10.1016/j.ijantimicag.2024.107425 39734051

[20] Helsens N, Sadek M, Le Terrier C et al Reduced susceptibility to aztreonam-avibactam conferred by acquired AmpC-type beta-lactamases in PBP3-modified Escherichia coli. Eur J Clin Microbiol Infect Dis 2024. 10.1007/s10096-024-04769-z Online ahead of print 38319508

[21] Livermore DM, Mushtaq S, Vickers A et al Activity of aztreonam/avibactam against metallo-beta-lactamase-producing enterobacterales from the UK: impact of penicillin-binding protein-3 inserts and CMY-42 beta-lactamase in Escherichia coli. Int J Antimicrob Agents 2023; 61: 106776. 10.1016/j.ijantimicag.2023.106776 36893810

[22] Long H, Zhao F, Feng Y et al Global emergence of Escherichia coli with PBP3 insertions. J Antimicrob Chemother 2025; 80: 178–81. 10.1093/jac/dkae393 39478333

[23] Haidar G, Kline EG, Kitsios GD et al Emergence of high-level aztreonam-avibactam and cefiderocol resistance following treatment of an NDM-producing Escherichia coli bloodstream isolate exhibiting reduced susceptibility to both agents at baseline. JAC Antimicrob Resist 2024; 6: dlae141. 10.1093/jacamr/dlae141 39239090 PMC11375572

[24] Chavda KD, Chen L, Fouts DE et al Comprehensive genome analysis of carbapenemase-producing Enterobacter spp.: new insights into phylogeny, population structure, and resistance mechanisms. mBio 2016; 7: e02093-16. 10.1128/mBio.02093-16 27965456 PMC5156309

[25] Halder G, Chaudhury BN, Mandal S et al Whole genome sequence-based molecular characterization of blood isolates of carbapenem-resistant Enterobacter cloacae complex from ICU patients in Kolkata, India, during 2017–2022: emergence of phylogenetically heterogeneous Enterobacter hormaechei subsp. xiangfangensis. Microbiol Spectr 2024; 12: e0352923. 10.1128/spectrum.03529-23 38385742 PMC10986559

[26] Kimbrough JH, Ewald JM, Karr M et al Enterobacter hormaechei carrying blaNDM predominates among Carbapenem-resistant Enterobacter spp. collected during the SENTRY antimicrobial surveillance program (2016–2021). *ECCMID* 2023. Copenhagen, Denmark, 2023. Poster #283.

